# Graphene H-Waveguide for Terahertz Lasing Applications: Electromagnetic Quasi-Linear Theory

**DOI:** 10.3390/nano10122415

**Published:** 2020-12-03

**Authors:** Guennadi A. Kouzaev

**Affiliations:** Department of Electronic Systems, Norwegian University of Science and Technology-NTNU, No-7491 Trondheim, Norway; guennadi.kouzaev@ntnu.no; Tel.: +47-735-96369

**Keywords:** graphene, terahertz, lasing, graphene H-waveguide

## Abstract

A novel graphene H-waveguide is proposed for active terahertz components. A graphene film illuminated by strong pumping light shorts the parallel conductor plates. The terahertz modes propagating along this film are amplified at certain conditions. A rigorous electromagnetic (EM) quasi-linear method of analytical calculations of ${\mathrm{TE}}_{y}$ and ${\mathrm{TM}}_{y}$ eigenmodes is used in this paper to select these conditions. Among them is the use of bound ${\mathrm{TE}}_{y}$ modes interacting with graphene plasmons at frequencies of negative graphene resistance, minimizing conductor loss associated with parallel plates, and excluding the current-crowding effect from the waveguide design. The limitations of the used theory are considered, and the applications of this waveguide are proposed.

## 1. Introduction

Today much attention is paid to two-dimensional materials and electronic components. Graphene is the first one consisting of carbon atoms and composing a flexible and stretchable honeycomb crystal lattice discovered by Geim and Novoselov [1]. The theory of this material is based on quantum physics, and it is rather complicated. For instance, the electricity motion is described with oscillation of electron plasma. Excited plasmons are coupled to electromagnetic (EM) waves, and even a single sheet or strip of graphene has waveguiding properties [2]. For electronic applications, a convenient parameter describing graphene is its conductivity $\sigma_{g}$, for which several approximate formulas are known [3,4,5,6,7,8].

The graphene sheets or strips can be placed over a dielectric substrate surface and combined with conductors forming the waveguides [2]. The EM mathematical analytical and numerical models of graphene transmission lines are published in many contributions, and they use the field-matching [7,8], transverse resonance [9], integral equation [10], and finite-difference or finite element methods [2,11].

A part of graphene’s unique properties is its controllability by electric, magnetic, light fields, and chemical doping [12,13]. The high electron mobility of graphene allows developing transistors of terahertz frequencies, including the components sensitive to optical irradiation. At a substantial incident EM field, graphene shows moderate nonlinearity of its conductivity, and the terahertz parametric amplifiers, modulators, etc. are known [14,15,16,17].

Interesting techniques are when the real part of graphene complex conductivity $\sigma_{g}$ is negative, and it is realized in the terahertz range by optical pumping or charge injection [18,19,20]. These graphene effects and the reached gain in order of 1000 ${\mathrm{cm}}^{-1}$ are confirmed by measurements or/and EM numerical simulations in several graphene-based structures. Among them are the graphene film or graphene strips over a dielectric sheet [5], metal/graphene strips over a dielectric substrate [21], conductor-backed graphene-slot waveguide [22], a dielectric cylinder covered by graphene [23], and hybrid graphene-dielectric microtube waveguides [24].

The effectiveness of optically induced or charge injection lasing can be enhanced using the multilayered graphene [22,25]. For instance, it allows decreasing the required intensity of light or shifting the negative conductivity towards the millimeter-wave frequencies with an overall increase in permitted currents in graphene [26]. The graphene-based components’ properties can be enhanced further by integrating the graphene layers with the semiconductor heterostructures [27,28,29,30].

The nearest competitors to the graphene-based devices are the resonant tunneling diodes showing the amplification and generation of terahertz irradiation [31,32]. A promising technology is with the terahertz discrete GaN components, including the negative resistance diodes [33].

**Figure 1:**
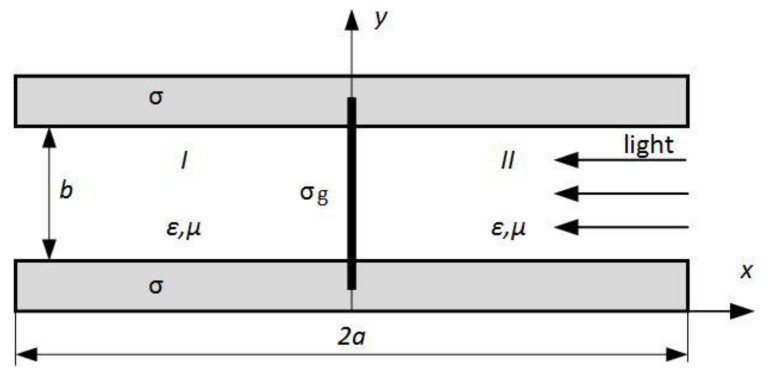
Cross-section of a graphene H-waveguide for lasing application. The terahertz waveguide modes propagate along the z-axis, which is normal to the picture plane.

In this contribution, a novel low-loss graphene-based waveguide (Figure 1) is proposed and analytically studied by a rigorous EM method. A parallel-plate waveguide is shorted by a graphene film uniformly illuminated by pumping light. This H-waveguide directs the EM modes with the amplification due to the induced negative graphene conductivity. The waveguide loss decrease is provided by excluding the sharp-edged graphene and conductor strips from this design, the employ of modes with the electric field decreasing towards the conductor plates, and by proper geometry of the waveguide, allowing better concentration of the modal field near the graphene film. The analytically calculated results show dependence of complex propagation constant and modal fields on the frequency, dielectric filling, waveguide geometry, and quasi-Fermi energy level defined by pumping light intensity.

## 2. Proposed Graphene Component, Its Theory, and Used Methods

The terahertz range of frequencies is the place of complicated wave and material effects, and the development of new components is a challenging process [34]. A part of the problems can be diminished by reducing the cross-section of elements and geometry tailored to the employed effects and component application.

In microwaves, an H-waveguide consisting of two parallel conductor plates and a dielectric insert forming a figure of the letter H is known. The main mode of this waveguide is the quasi-TEM (transverse-EM) one [35] with $E_{y}$ (“vertical”) field component. An increased concentration of the field inside of this dielectric sheet was found. Later, this waveguide was proposed for the millimeter-wave range, called the nonradiative dielectric (NRD) waveguide [36]. This waveguide’s used mode is the lowest ${\mathrm{TE}}_{y}^{\left(1\right)}$ one. It has a relatively low conductor loss due to the electric field’s transverse orientation parallel to the conductor plates. The irradiation of this insert in the transverse directions is reduced due to the waveguide’s small height preventing propagation of this mode away from this dielectric sheet. Some terahertz applications of this waveguide are described in Refs. [36,37,38,39], for instance.

In this paper, the central dielectric sheet of a nonradiative dielectric waveguide is substituted by a single or multilayered graphene film (Figure 1, black-colored vertical strip), shorting the conductor plates. Similar to the mentioned prototype NRD, the ${\mathrm{TE}}_{y}^{\left(m\right)}$ modes are proposed for signaling to decrease the plate-associated loss. The height of this waveguide is chosen to prevent the propagation of these modes without graphene insert. It allows reducing the irradiation towards the transverse ±x− directions. This proposed waveguide is convenient for the pumping light illumination of graphene from its left or right side to make conductivity Re(σg) negative over a specific frequency. Besides, this structure is pertinent for charge injection to graphene by applying a DC voltage to the waveguide’s conductor plates and realizing its associated amplification. This design has no graphene or conductor strips at the difference to known structures, causing an essential loss due to the current-crowding effect [8,40] near the mentioned sharp edges strips. Today, the packaging technologies exist, allowing the manufacturing of vertically oriented graphene structures of this type. For instance, Ref. [41] is used to make the graphene strips suspended over-the-trenches.

### 2.1. Hybrid Mode Theory

Here, this waveguide’s theory in the quasi-linear approximation is given, i.e., supposing that the weak signal terahertz field does not influence graphene’s conductivity. Otherwise, the nonlinear methods of Maxwell equations solution should be applied [14,15]. Besides, it is supposed that a plane pumping light wave illuminates the graphene film, and it has a uniform conductivity distribution along its surface.

In general, due to graphene conductivity’s anisotropy ${\underset{↔}{\sigma }}_{g}$, the waveguide’s modal field should be hybrid with all six components. Assuming the time dependence as $e^{j\omega t}$, we introduce two vector potential functions [42,43] in each cross-section of the domains I and II (Figure 1)

(1) $$\begin{array}{l} F^{\left(I,II\right)}\left(x,y,z\right)=y_{0}F_{y}^{\left(I,II\right)}\left(x,y\right)e^{-jk_{z}z},\\ A^{\left(I,II\right)}\left(x,y,z\right)=y_{0}A_{y}^{\left(I,II\right)}\left(x,y\right)e^{-jk_{z}z} \end{array}$$

where $y_{0}$ is the vector unit, j is the imaginary unit, and $k_{z}$ is the longitudinal propagation constant, which is the subject to be obtained.

The four vector field components needed for further treatments in each domain are

(2) $$\begin{array}{l} H_{y}^{\left(I,II\right)}=-\frac{j}{\omega \varepsilon \mu }\left(\frac{\partial^{2}}{\partial y^{2}}+\kappa^{2}\right)F_{y}^{\left(I,II\right)}\left(x,y\right)e^{-jk_{z}z},\;H_{z}^{\left(I,II\right)}=\left(-\frac{k_{z}}{\omega \varepsilon \mu }\frac{\partial F_{y}^{\left(I,II\right)}\left(x,y\right)}{\partial y}+\frac{1}{\mu }\frac{\partial A_{y}^{\left(I,II\right)}\left(x,y\right)}{\partial x}\right)e^{-jk_{z}z},\\ E_{y}^{\left(I,II\right)}=-\frac{j}{\omega \mu \varepsilon }\left(\frac{\partial^{2}}{\partial y^{2}}+\kappa^{2}\right)A_{y}^{\left(I,II\right)}\left(x,y\right)e^{-jk_{z}z},\;E_{z}^{\left(I,II\right)}=-\left(\frac{1}{\varepsilon }\frac{\partial F_{y}^{\left(I,II\right)}\left(x,y\right)}{\partial x}+\frac{k_{z}}{\omega \mu \varepsilon }\frac{\partial A_{y}^{\left(I,II\right)}\left(x,y\right)}{\partial y}\right)e^{-jk_{z}z} \end{array}$$

where ω=2πf and f are the cycling and driving frequencies, correspondingly, $\mu =\mu_{0}\mu_{r}$ with $\mu_{0}$ and $\mu_{r}$ are the absolute vacuum and substrate relative permeabilities, correspondingly, $\varepsilon =\varepsilon_{0}\varepsilon_{r}$ where $\varepsilon_{0}$ and $\varepsilon_{r}$ are the absolute vacuum and substrate relative permittivities, correspondingly, and $\kappa^{2}=k_{0}^{2}\varepsilon_{r}\mu_{r}$, $k_{0}=\omega /c$, and c is the light velocity in vacuum.

The mode-matching method is used to solve this boundary value problem. In each geometry domain I or II (Figure 1), a set of discrete modes of an ideal parallel-plate waveguide is written. Taking into account the uniform geometry of graphene film and its constant conductivity distribution along the y−axis, the radiation continuous-spectrum field is omitted [36,44].

The vector potentials for these modes are

(3) $$\begin{array}{l} F_{m}^{\left(I\right)}\left(x,y,z\right)=y_{0}F_{m}^{\left(I\right)}\sin\left(k_{y}^{\left(m\right)}y\right)e^{jk_{x}^{\left(m\right)}x}e^{-jk_{z}^{\left(m\right)}z},\\ F_{m}^{\left(II\right)}\left(x,y,z\right)=y_{0}F_{m}^{\left(II\right)}\sin\left(k_{y}^{\left(m\right)}y\right)e^{-jk_{x}^{{}^{\left(m\right)}}x}e^{-jk_{z}^{\left(m\right)}z},\\ A_{m}^{\left(I\right)}\left(x,y,z\right)=y_{0}A_{m}^{\left(I\right)}\cos\left(k_{y}^{\left(m\right)}y\right)e^{jk_{x}^{\left(m\right)}x}e^{-jk_{z}^{\left(m\right)}z},\\ A_{m}^{\left(II\right)}\left(x,y,z\right)=y_{0}A_{m}^{\left(II\right)}\cos\left(k_{y}^{\left(m\right)}y\right)e^{-jk_{x}^{\left(m\right)}x}e^{-jk_{z}^{\left(m\right)}z} \end{array}$$

where $F_{m}^{\left(I,II\right)}$ and $A_{m}^{\left(I,II\right)}$ are the unknown potential function amplitudes, $k_{y}=m\pi /b$, m is the modal number, and ${\left(k_{z}^{\left(m\right)}\right)}^{2}=\kappa^{2}-{\left(k_{x}^{\left(m\right)}\right)}^{2}-{\left(k_{y}^{\left(m\right)}\right)}^{2}$. It is seen that each vector potential function in (3) satisfies the radiation boundary conditions at x=∓∞, and the modes can be leaky at certain conditions [45,46].

Supposing an infinitely thin graphene layer (Figure 1), the modal fields should be matched at x=0 using the following boundary conditions

(4) $${\underset{↔}{\sigma }}_{g}E_{m,\tau }^{\left(I\right)}=x_{0}\times \left(H_{m,\tau }^{\left(II\right)}-H_{m,\tau }^{\left(I\right)}\right)$$

(5) $$E_{m,\tau }^{\left(II\right)}-E_{m,\tau }^{\left(I\right)}=0,$$

where $E_{m,\tau }^{\left(I,II\right)}$ and $H_{m,\tau }^{\left(I,II\right)}$ are the field vectors tangential to the plane x=0, y,z. It provides four linear algebraic homogeneous equations regarding $F_{m}^{\left(I,II\right)}$ and $A_{m}^{\left(I,II\right)}$. Solving (4) and (5), i.e., calculating a zero of the system’s determinant allows us to obtain a complex propagation constant $k_{z}^{\left(m\right)}$ from, unfortunately, a transcendental equation in this hybrid mode case.

### 2.2. $TE_{y}^{\left(m\right)}$ Modes

The scalar approximation of graphene’s conductivity allows separating the hybrid modes into two types [42,43] and simplifying the solution.

Consider the treatment of the ${\mathrm{TE}}_{y}^{\left(m\right)}$ modes. The needed tangential-to-graphene field components are

(6) $$\begin{array}{l} H_{y,m}^{\left(I,II\right)}\left(x,y,z\right)=-\frac{j}{\omega \mu \varepsilon }\left(\frac{\partial^{2}}{\partial y^{2}}+\kappa^{2}\right)F_{y,m}^{\left(I,II\right)}\left(x,y\right)e^{-jk_{z}^{\left(m\right)}z},\;\\ H_{z,m}^{\left(I,II\right)}\left(x,y,z\right)=-\frac{k_{z}^{\left(m\right)}}{\omega \mu \varepsilon }\frac{\partial F_{y,m}^{\left(I,II\right)}\left(x,y\right)}{\partial y}e^{-jk_{z}^{\left(m\right)}z},\\ E_{y,m}^{\left(I,II\right)}\left(x,y,z\right)=0,\;E_{z,m}\left(x,y,z\right)=-\frac{1}{\varepsilon }\frac{\partial F_{y,m}^{\left(I,II\right)}\left(x,y\right)}{\partial x}e^{-jk_{z}^{\left(m\right)}z} \end{array}$$

where

(7) $$\begin{array}{l} F_{y,m}^{\left(I\right)}\left(x,y\right)=F_{m}^{\left(I\right)}\sin\left(k_{y}^{\left(m\right)}y\right)e^{jk_{x}^{\left(m\right)}x},\\ F_{y,m}^{\left(II\right)}\left(x,y\right)=F_{m}^{\left(II\right)}\sin\left(k_{y}^{\left(m\right)}y\right)e^{-jk_{x}^{\left(m\right)}x}. \end{array}$$

Using the field components (6), the boundary conditions (4) and (5) at x=0 are satisfied. It gives a system of two linear homogeneous equations regarding $F_{m}^{\left(I\right)}$ and $F_{m}^{\left(II\right)}$. Zeroing these equations’ determinant yields a simple formula for the modal propagation constant $k_{z}^{\left(m\right)}$

(8) $$\begin{array}{c} {\left(k_{z}^{\left(m\right)}\right)}^{2}=\left(\kappa^{2}-{\left(k_{y}^{\left(m\right)}\right)}^{2}\right)\left[1-\frac{4\left(\kappa^{2}-{\left(k_{y}^{\left(m\right)}\right)}^{2}\right)}{{\left(\omega \mu \sigma_{g}\right)}^{2}}\right],\\ m=1,2,3,\ldots ,\infty . \end{array}$$

This expression gives a pair of forward and backward modes [47] whose propagation constants $k_{z}^{\left(m\right)}$ are different only by a sign before the root of (8).

The found longitudinal modal constant $k_{z}^{\left(m\right)}$ allows calculating the lateral propagation number $k_{x}^{\left(m\right)}=\mp \sqrt{k_{0}^{2}\varepsilon_{r}\mu_{r}-{\left(k_{y}^{\left(m\right)}\right)}^{2}-{\left(k_{z}^{\left(m\right)}\right)}^{2}}$. In lossy/active open waveguides, it gives a pair of modes of untrivial difference between them, which will be studied below.

### 2.3. $TM_{y}^{\left(m\right)}$ Modes

The ${\mathrm{TM}}_{y}^{\left(m\right)}$ modes are handled similarly. The needed for further treatment field components are:

(9) $$\begin{array}{l} H_{y,m}^{\left(I,II\right)}\left(x,y,z\right)=0,\;H_{z,m}^{\left(I,II\right)}\left(x,y,z\right)=\frac{1}{\mu }\frac{\partial A_{y,m}\left(x,y\right)}{\partial x}e^{-jk_{z}^{\left(m\right)}z},\\ E_{y,m}^{\left(I,II\right)}\left(x,y,z\right)=-\frac{j}{\omega \mu \varepsilon }\left(\frac{\partial^{2}}{\partial y^{2}}+\kappa^{2}\right)A_{y,m}\left(x,y\right)e^{-jk_{z}^{\left(m\right)}z},\\ E_{z,m}^{\left(I,II\right)}\left(x,y,z\right)=-\frac{k_{z}^{\left(m\right)}}{\omega \mu \varepsilon }\frac{\partial A_{y,m}\left(x,y\right)}{\partial y}e^{-jk_{z}^{\left(m\right)}z} \end{array}$$

where

(10) $$\begin{array}{l} A_{y,m}^{\left(I\right)}\left(x,y\right)=A_{m}^{\left(I\right)}\cos\left(k_{y}^{\left(m\right)}y\right)e^{jk_{x}^{\left(m\right)}x},\\ A_{y,m}^{\left(II\right)}\left(x,y\right)=A_{m}^{\left(II\right)}\cos\left(k_{y}^{\left(m\right)}y\right)e^{-jk_{x}^{\left(m\right)}x}. \end{array}$$

Using (4), (5), (9) and (10), an analytical formula is derived for the modal propagation constants $k_{z}^{\left(m\right)}$ of the ${\mathrm{TM}}_{y}^{\left(m\right)}$ modes

(11) $$\begin{array}{l} {\left(k_{z}^{\left(m\right)}\right)}^{2}=\left(\kappa^{2}-{\left(k_{y}^{\left(m\right)}\right)}^{2}\right)\left[1-{\left(\frac{\sigma_{g}}{2\omega \varepsilon }\right)}^{2}\left(\kappa^{2}-{\left(k_{y}^{\left(m\right)}\right)}^{2}\right)\right],\\ m=0,1,2,\ldots ,\infty . \end{array}$$

Similarly to the ${\mathrm{TE}}_{y}$ solutions, there will be forward and backward modes. Each of these modes is paired due to the root $k_{x}^{\left(m\right)}=\mp \sqrt{k_{0}^{2}\varepsilon_{r}\mu_{r}-{\left(k_{y}^{\left(m\right)}\right)}^{2}-{\left(k_{z}^{\left(m\right)}\right)}^{2}}$.

## 3. Results and Discussion

### 3.1. Graphene Conductivity

A graphene conductivity formula used here is from Refs. [4,18]

(12) $$\sigma_{g}=\left(\frac{e^{2}}{4\hbar }\right)\left\{\frac{8k_{B}T\tau }{\pi \hbar \left(1-j\omega \tau \right)}\ln\left[1+\exp\left(\frac{E_{F}}{k_{B}T}\right)\right]+\tanh\left(\frac{\hbar \omega -2E_{F}}{4k_{B}T}\right)-\frac{4\hbar \omega }{j\pi }\int_{0}^{\infty }\frac{G\left(E-E_{F}\right)-G\left(\frac{\hbar \omega }{2},E_{F}\right)}{{\left(\hbar \omega \right)}^{2}-4E^{2}}dE\right\},$$

where e is the electron charge, ℏ is the reduced Planck’s constant, $k_{B}$ is the Boltzmann constant, T is the temperature in Kelvin, τ is the graphene relaxation time, ω is the terahertz cycling frequency, $E_{F}$ is the quasi-Fermi energy, and $G\left(E,E^{\prime }\right)=\frac{\sinh\left(E/k_{B}T\right)}{\cosh\left(E/k_{B}T\right)+\cosh\left(E^{\prime }/k_{B}T\right)}$. In this formula, the level of quasi-Fermi energy is defined by pumping light.

**Figure 2:**
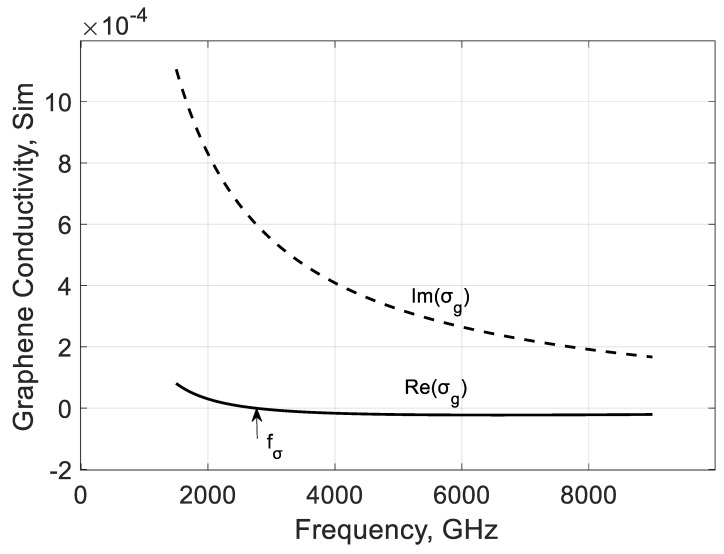
Frequency dependencies of real (solid line) and imaginary (dash line) parts of graphene conductivity $\sigma_{g}$. Parameters used for calculations: $E_{F}=40\;\mathrm{meV}$, T=300 K, and τ=1 ps. Parameter data are from Ref. [21].

Figure 2 illustrates the frequency dependence of graphene conductivity’s real and imaginary parts $\sigma_{g}$, denoting a frequency point Re(σg(fσ))=0.

The zero position of Re(σg) on the frequency axis depends on the infrared pumping light defining the quasi-Fermi level in (12). If graphene is in its equilibrium state, the carrier distribution is described by a single Fermi energy level depending on the graphene properties and temperature. Light illumination of graphene leads to a new dynamic stage of charge carrier distribution characterized by the quasi-Fermi energy levels for electrons $E_{F_{n}}$ and holes $E_{F_{p}}$. Proper calculation of these levels requires solving an ab initio problem or using semiclassical equations [48] for charge transport in graphene.

In practice, according to the data of different authors, it is supposed that for the charge densities in order of $10^{10}–10^{11}{\mathrm{cm}}^{-2}$, light wavelength around 0.8–10 μm, and source intensity in order 2–$2000\;W/{\mathrm{cm}}^{2}$, the quasi-Fermi energy level $E_{F}=E_{F_{n}}=E_{F_{p}}$ may vary in the limits 20–150 meV [19,22,27]. These values of $E_{F}$ are used in this paper for the EM calculation of the proposed waveguide. It is estimated that the amplified signals in the quasi-linear regime can have a level of 10 µW for the diffraction type of devices [21]. The gain factor can reach the values of the order 1000 ${\mathrm{cm}}^{-1}$, and it can be increased further using the multilayer graphene films and graphene-semiconductor heterostructures.

The photo-induced carriers pump energy to traveling plasmons of the terahertz range, and the EM coupled-wave is amplified. This effect is approximately described by a graphene conductivity Formula (12) obtained using Kubo’s approach. In Ref. [18], it is shown that wave amplification occurs at frequencies over $f_{\sigma }$ where Re(σg)<0.

### 3.2. TE_y_ Longitudinal Propagation Constant

Analyzing Formulas (8) and (11), it is seen that a critical frequency $f_{c}^{\left(m\right)}$ exists where $\kappa^{2}-{\left(k_{y}^{\left(m\right)}\right)}^{2}=0$ for m≥1. The wave propagation below this frequency is still supported by graphene film’s guiding properties, although the parallel-plate waveguide does not propagate its corresponding volumetrical mode.

**Figure 3:**
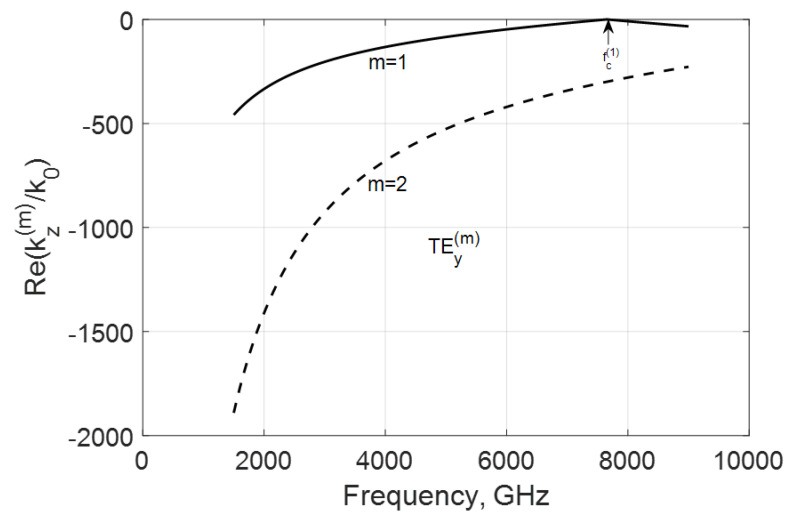
Normalized real parts of the modal propagation constants $k_{z}^{\left(m\right)}/k_{0}$ of the first two ${\mathrm{TE}}_{y}^{\left(m\right)}$ backward modes of a graphene H-waveguide of the height b=0.01 mm, width 2a=∞, $\varepsilon_{r}=3.84\;$, and $\;\mu_{r}=1$. Parameters used for calculation of $\sigma_{g}$ are from Ref. [21]: T=300 K, $E_{F}=40\;\mathrm{meV}$, and τ=1 ps.

**Figure 4:**
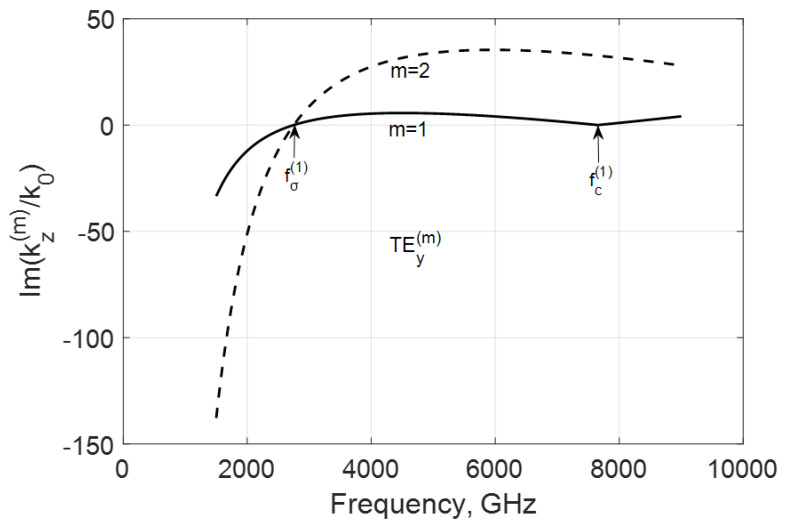
Normalized imaginary parts of the propagation constant Im(kz(m)/k0) of the first two ${\mathrm{TE}}_{y}^{\left(m\right)}$ backward modes of a graphene H-waveguide of the height b=0.01 mm and width 2a=∞, $\varepsilon_{r}=3.84$, and $\;\mu_{r}=1$. Parameters used for calculation of $\sigma_{g}$ are from Ref. [21]: T=300 K, $E_{F}=40\;\mathrm{meV}$, and τ=1 ps.

In Figure 3 and Figure 4, the frequency dependencies of the normalized real and imaginary parts of the propagation constant $k_{z}^{\left(m\right)}/k_{0}=-\left(\beta^{\left(m\right)}+j\alpha^{\left(m\right)}\right)/k_{0}$ of the first two ${\mathrm{TE}}_{y}^{\left(m\right)}$ modes are shown for the case when $f_{\sigma }<f_{c}^{\left(1\right)}$. These real parts are negative for the backward modes before and after critical frequencies $f_{c}^{\left(m\right)}$. Because these modes are coupled to graphene with its high specific conductivity, they are very slow and have increased phase constants $\beta_{m}$.

The simulation of the imaginary parts of propagation constants shows that the negative conductivity effect makes these modes propagate with amplification occurring below and over their critical frequencies $f_{c}^{\left(m\right)}$ because there Im(kz(m)/k0)>0 (Figure 4). Remarkably, this value is growing with the modal number in the considered range of frequencies. An explanation is with the coupling of the modal field to graphene and the plasmon-surface nature of these modes. With the modal number m, the field localizes near the film and the modal properties are defined by graphene more strongly.

**Figure 5:**
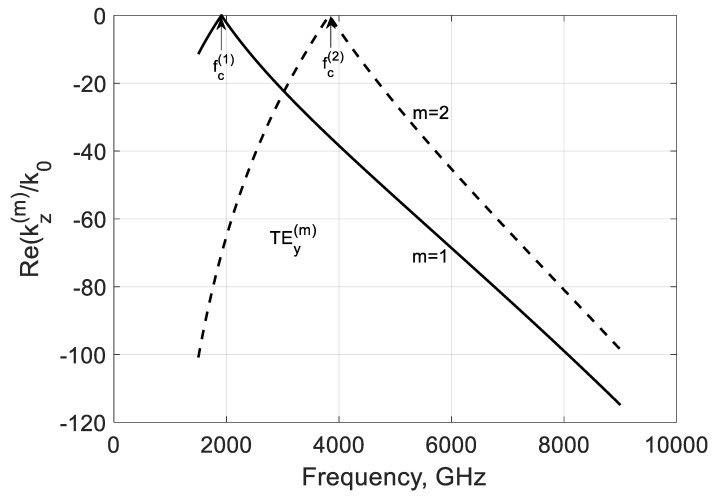
Normalized real parts of the modal propagation constants $k_{z}^{\left(m\right)}/k_{0}$ of the first two ${\mathrm{TE}}_{y}^{\left(m\right)}$ backward modes of a graphene H-waveguide of the height b=0.04 mm, width 2a=∞, $\varepsilon_{r}=3.84\;$, and $\;\mu_{r}=1$. Parameters used for calculation of $\sigma_{g}$ are from Ref. [21]: T=300 K, $E_{F}=40\;\mathrm{meV}$, and τ=1 ps.

**Figure 6:**
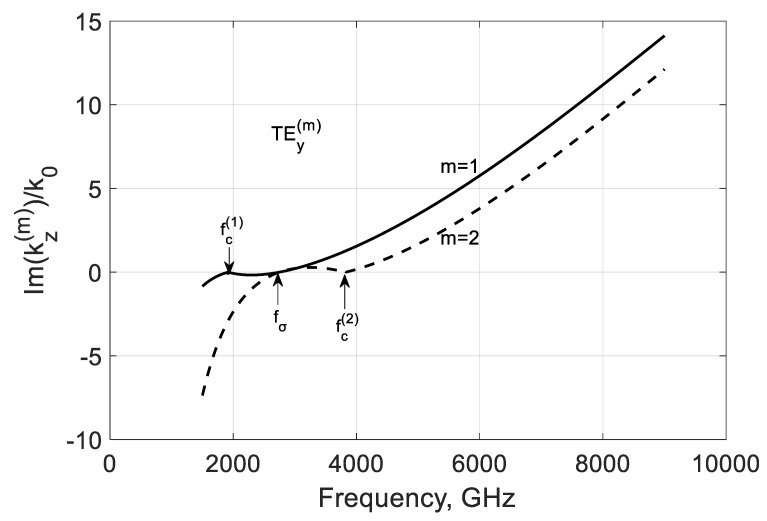
Normalized imaginary parts of the propagation constant Im(kz(m)/k0) of the first two ${\mathrm{TE}}_{y}^{\left(m\right)}$ backward modes of a graphene H-waveguide of the height b=0.04 mm and width 2a=∞,$\varepsilon_{r}=3.84$, and $\;\mu_{r}=1$. Parameters used for calculation of $\sigma_{g}$ are from Ref. [21]: T=300 K, $E_{F}=40\;\mathrm{meV}$, and τ=1 ps.

Another variant is when $f_{c}^{\left(1\right)}<f_{\sigma }$, which can be reached by increasing the waveguide height b, for instance. In this case, both modes have critical frequencies $f_{c}^{\left(m\right)}$ in the considered range (Figure 5 and Figure 6). These waves are amplified over frequency $f_{\sigma }$, which is typical for both modes, but, here, the amplification is more substantial for the lowest ${\mathrm{TE}}_{y}^{\left(1\right)}$ mode. The increase in Im(kz(m)/k0) over the frequencies $f_{c}^{\left(m\right)}$ is explained by the decrease in the shortening effect of graphene film (see Figure 2).

### 3.3. Study of the Bound and Leaky Regimes of the TE_y_ and TM_y_ Modes

As mentioned in Section 2, each forward or backward mode has a paired solution $k_{x}^{\left(m\right)}=\mp \sqrt{k_{0}^{2}\varepsilon_{r}\mu_{r}-{\left(k_{y}^{\left(m\right)}\right)}^{2}-{\left(k_{z}^{\left(m\right)}\right)}^{2}}$. In open structures and lossy material parameters, these modes can be registered in the bound or leaky states [46]. In graphene-based waveguides, the bound or leaky regimes depend on graphene parameters, surrounding material, and waveguide housing. The same mode can be bounded or leaky at different frequencies, and there is a need to study the frequency dependence of the lateral propagation constant $k_{x}$ to define these states.

**Figure 7:**
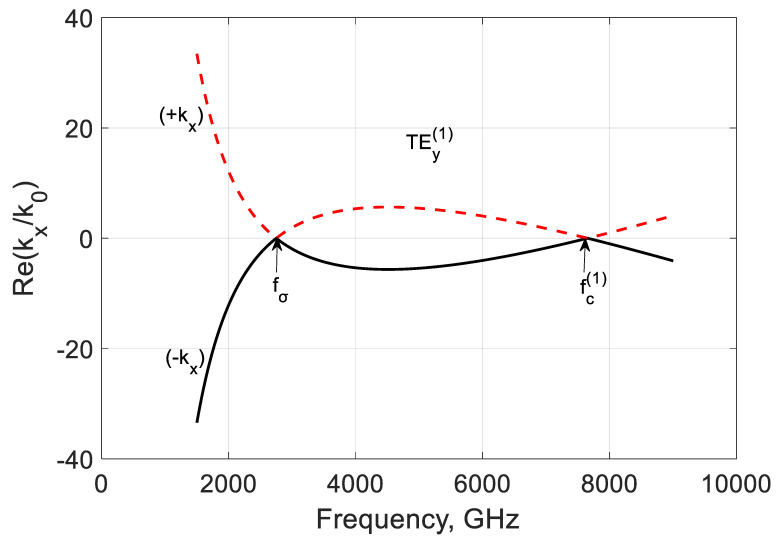
Normalized real parts of $\mp k_{x}/k_{0}$ of the ${\mathrm{TE}}_{y}^{\left(1\right)}$ backward mode of a graphene H-waveguide of the height b=0.01 mm and width 2a=∞, $\varepsilon_{r}=3.84$, and $\;\mu_{r}=1$. Parameters used for calculation of $\sigma_{g}$ are from Ref. [21]: T=300 K, $E_{F}=40\;\mathrm{meV}$, and τ=1 ps.

Figure 7 shows the real parts of this propagation constant $k_{x}^{\left(1\right)}=\mp \sqrt{k_{0}^{2}\varepsilon_{r}\mu_{r}-{\left(k_{y}^{\left(1\right)}\right)}^{2}-{\left(k_{z}^{\left(1\right)}\right)}^{2}}$. The modes are marked according to the sign before the root as $\left(-k_{x}\right)$ or $\left(+k_{x}\right)$. It is seen that this wavenumber part is influenced by the conductivity frequency behavior and critical modal effect.

**Figure 8:**
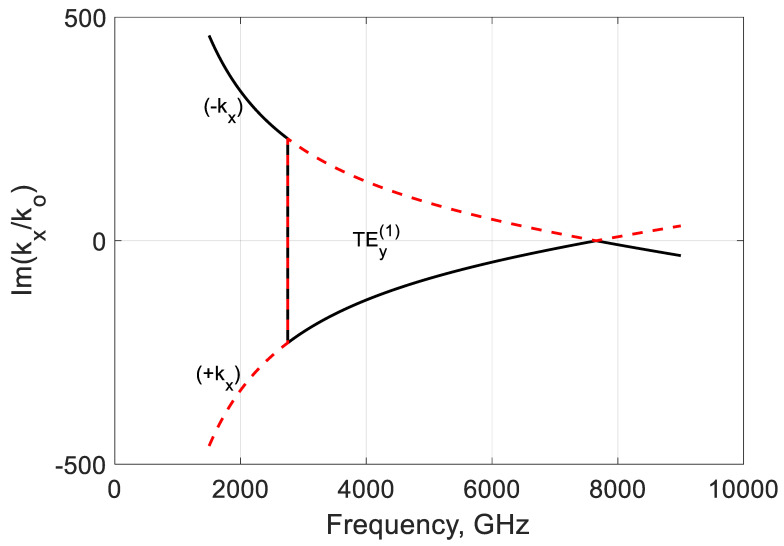
Normalized imaginary part of $\mp k_{x}/k_{0}$ of the ${\mathrm{TE}}_{y}^{\left(1\right)}$ backward mode of a graphene H-waveguide of the height b=0.01 mm and width 2a=∞, $\varepsilon_{r}=3.84$, and $\;\mu_{r}=1$. Parameters used for calculation of $\sigma_{g}$ are from Ref. [21]: T=300 K, $E_{F}=40\;\mathrm{meV}$, and τ=1 ps.

Figure 8 shows more information on the lateral properties of modes. At frequencies over $f_{\sigma }$, the imaginary part of $k_{x}$ for the mode marked $\left(-k_{x}\right)$ is negative; it corresponds to an exponential decrease in the field away from the graphene film at both ∓x− directions. At the same frequencies, the mode can be amplified along the z-axis due to the negative conductivity (Figure 4). Below $f_{\sigma }$, Im(kx(1)/k0)>0 and the field is exponentially growing with |x|. It means that in these frequencies, the mode is leaky, and it needs a special treatment to handle this unphysical solution [45,46,49,50]. It is not considered here because it is out of the paper’s scope.

Another mode marked as $\left(+k_{x}\right)$ is leaky strictly at the frequencies over $f_{\sigma }$, but it can be bounded in the lateral directions below the mentioned frequency. Here, the mode has an increased loss coupled with graphene (Figure 4). In this way, the H-waveguides show some self-filtration properties essential in the signal guiding and lasing.

**Figure 9:**
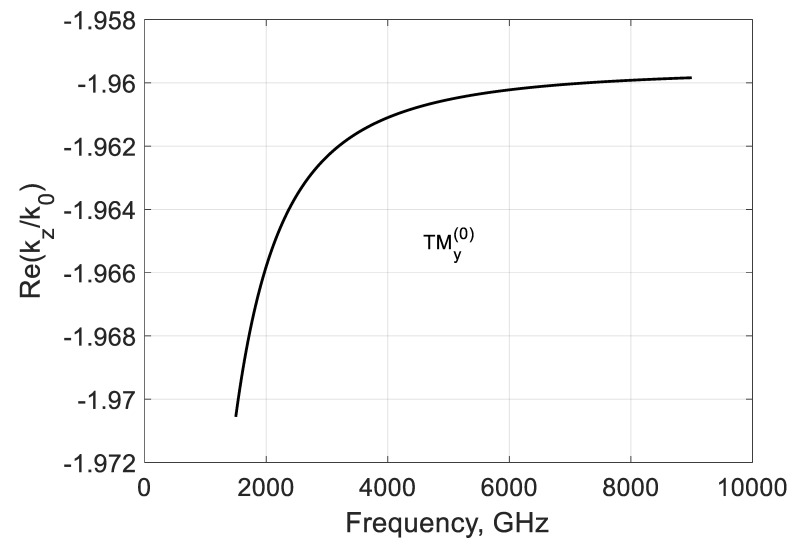
Normalized real part of the modal propagation constants $k_{z}^{\left(0\right)}/k_{0}$ of the first ${\mathrm{TM}}_{y}^{\left(0\right)}$ backward mode of a graphene H-waveguide of the height b=0.01 mm, width 2a=∞, $\varepsilon_{r}=3.84\;$, and $\;\mu_{r}=1$. Parameters used for calculation of $\sigma_{g}$ are from Ref. [21]: T=300 K, $E_{F}=40\;\mathrm{meV}$, and τ=1 ps.

For comparison, consider the backward ${\mathrm{TM}}_{y}^{\left(0\right)}$ mode (Section 2.3) and calculate the propagation constants $k_{z}^{\left(0\right)}$ and $k_{x}^{\left(0\right)}=\mp \sqrt{k_{0}^{2}\varepsilon_{r}\mu_{r}-{\left(k_{z}^{\left(0\right)}\right)}^{2}}$. It is seen from Figure 9 that this mode is fast and weakly coupled to graphene at all frequencies. This weak coupling is explained by the shortening of parallel plates by graphene film. 

**Figure 10:**
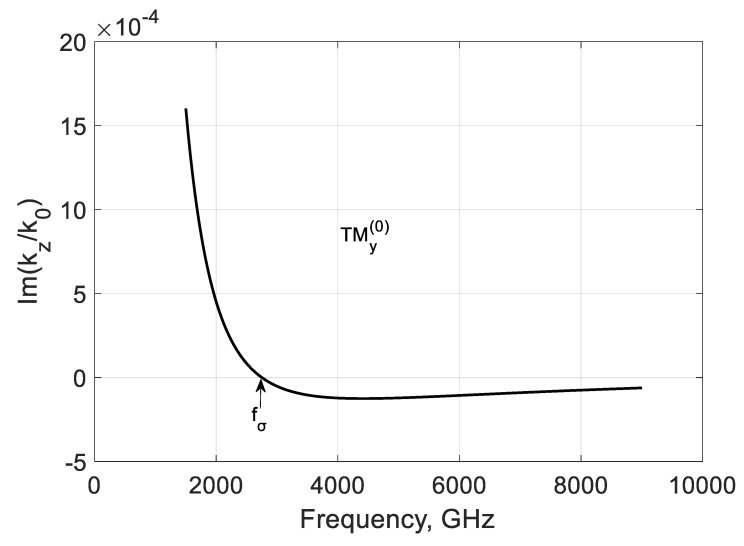
Normalized imaginary part of the modal propagation constants $k_{z}^{\left(0\right)}/k_{0}$ of the first ${\mathrm{TM}}_{y}^{\left(0\right)}$ backward mode of a graphene H-waveguide of the height b=0.01 mm, width 2a=∞, $\varepsilon_{r}=3.84\;$, and $\;\mu_{r}=1$. Parameters used for calculation of $\sigma_{g}$ are from Ref. [21]: T=300 K, $E_{F}=40\;\mathrm{meV}$, and τ=1 ps.

**Figure 11:**
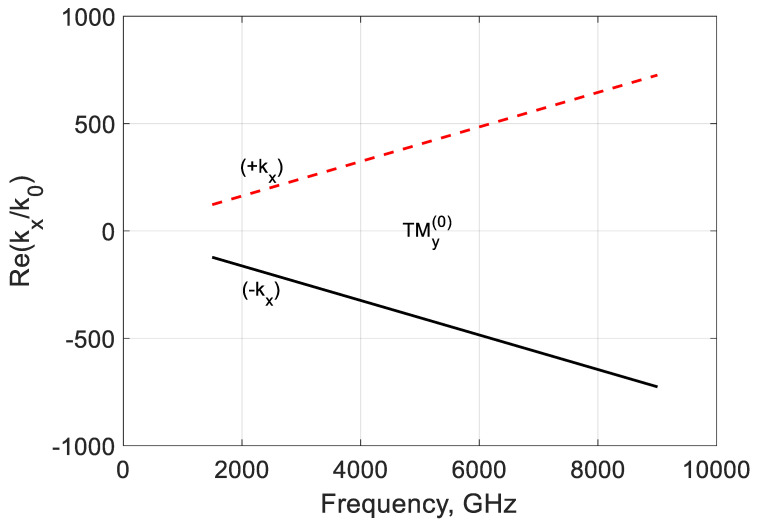
Normalized real part of the modal propagation constants $\mp k_{x}^{\left(0\right)}/k_{0}$ of the first ${\mathrm{TM}}_{y}^{\left(0\right)}$ backward mode of a graphene H-waveguide of the height b=0.01 mm, width 2a=∞, $\varepsilon_{r}=3.84\;$, and $\;\mu_{r}=1$. Parameters used for calculation of $\sigma_{g}$ are from Ref. [21]: T=300 K, $E_{F}=40\;\mathrm{meV}$, and τ=1 ps.

**Figure 12:**
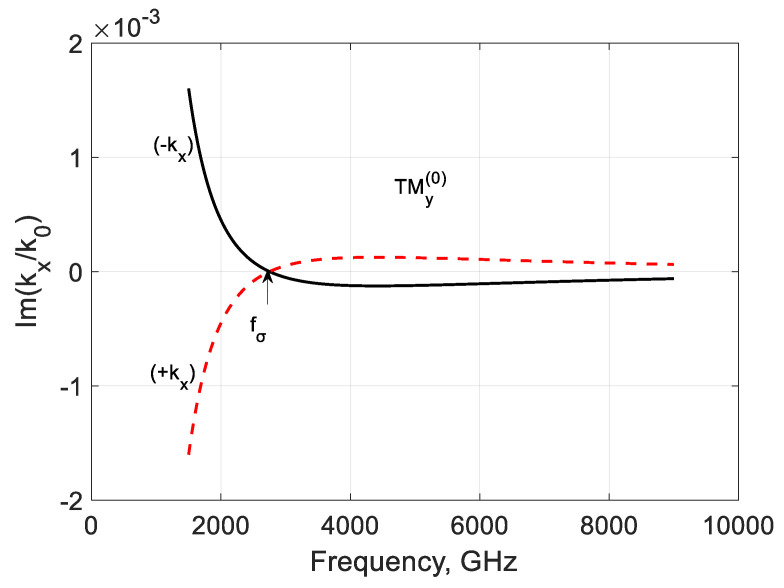
Normalized imaginary parts of the modal propagation constants $\mp k_{x}^{\left(0\right)}/k_{0}$ of the first ${\mathrm{TM}}_{y}^{\left(0\right)}$ backward mode of a graphene H-waveguide of the height b=0.01 mm, width 2a=∞, $\varepsilon_{r}=3.84\;$, and $\;\mu_{r}=1$. Parameters used for calculation of $\sigma_{g}$ are from Ref. [21]: T=300 K, $E_{F}=40\;\mathrm{meV}$, and τ=1 ps.

The imaginary part of the longitudinal propagation constant (Figure 10) is negative over $f_{\sigma }$, and the mode is damped. It may have a weak amplification at low frequencies because Im(kz(0)/k0)>0, but the modal localization should be studied. Figure 11 and Figure 12 show the frequency dependencies of the lateral propagation’s real and imaginary parts constant $k_{x}$. The mode marked as $\left(-k_{x}\right)$ is leaky below $f_{\sigma }$ and can be slightly amplified. This regime is interesting for active antenna applications. Over this graphene frequency, this mode is bounded but lossy. The mode marked as $\left(+k_{x}\right)$ is bounded and amplified at frequencies $f<f_{\sigma }$ but leaky and lossy at frequencies $f>f_{\sigma }$.

In this way, other ${\mathrm{TM}}_{y}$ modes can be studied. The modeling performed here shows the very weak coupling of these modes to graphene and low values of their normalized propagation constants compared to the ${\mathrm{TE}}_{y}$ modes. Then, the first ones can be considered as the higher-order modes.

Concluding the research with the modes of different polarizations, it is seen that their interactions with graphene films highly depend on the modal type, as it was shown earlier in [45] in the research on the Green function in graphene structures.

**Figure 13:**
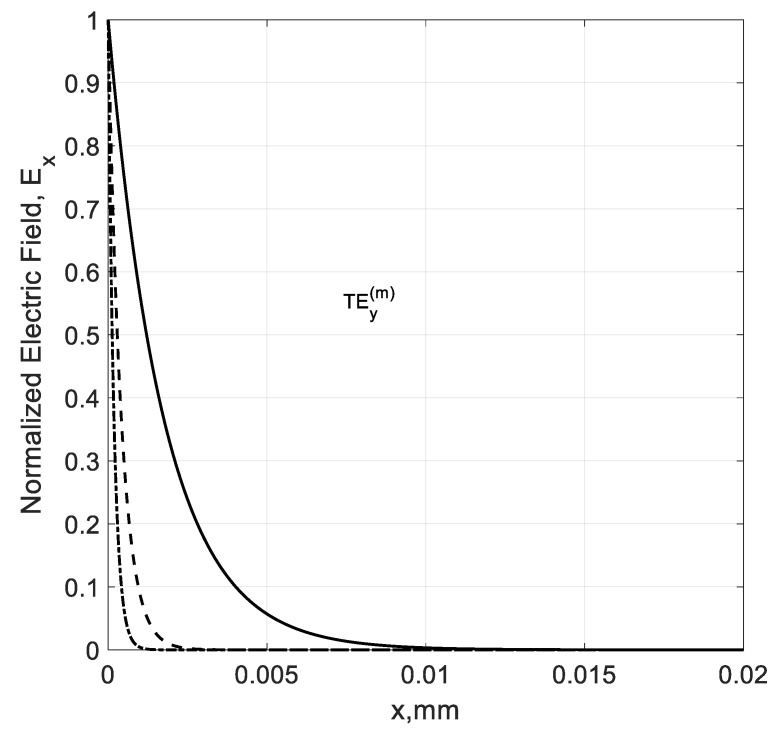
Normalized modal fields of the ${\mathrm{TE}}_{y}^{\left(m\right)}$ backward modes in the right part of a graphene H-waveguide (Figure 1) of the height b=0.01 mm and width 2a=∞, $\varepsilon_{r}=1$, and $\;\mu_{r}=1$ calculated at frequency f=4000 GHz. Solid line: m=1; dash line: m=2; and dash-dot line: m=3. Parameters used for calculation of $\sigma_{g}$ are from Ref. [21]: T=300 K, $E_{F}=40\;\mathrm{meV}$, and τ=1 ps.

Coming back to the ${\mathrm{TE}}_{y}^{\left(m\right)}$ modes, we can see from Figure 13 that the field calculation confirms the localized nature of proper ${\mathrm{TE}}_{y}^{\left(m\right)}$ modes at frequencies $f_{c}^{\left(m\right)}<f>f_{\sigma }$. The exponential field localization increases with the modal number m because the higher-order modes are more tightly coupled to graphene.

### 3.4. Controlling the Modes Varying the Parameters of Graphene H-Waveguides

**Figure 14:**
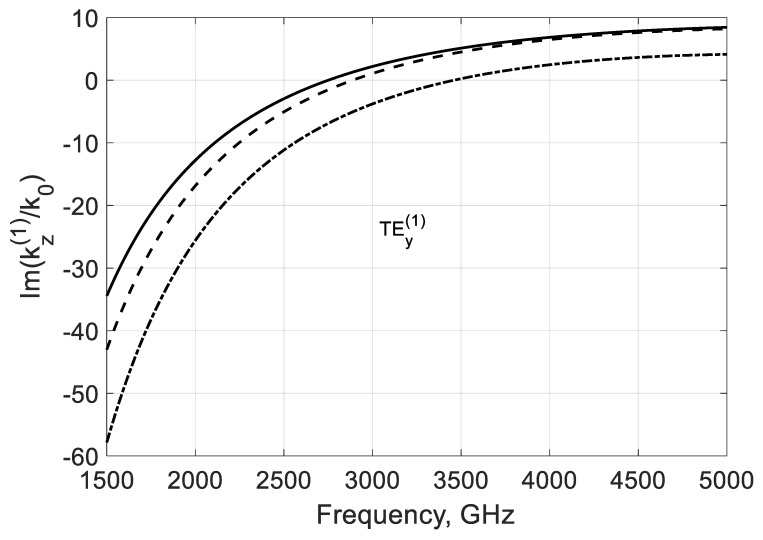
Influence of the quasi-Fermi energy level $E_{F}$ on the normalized imaginary part of the modal propagation constant $k_{z}^{\left(1\right)}/k_{0}$ of the ${\mathrm{TE}}_{y}^{\left(1\right)}$ backward mode calculated for a graphene H-waveguide of the height b=0.01 mm, width 2a=∞, $\varepsilon_{r}=1$, and $\;\mu_{r}=1$. Solid line: $E_{F}=40\;\mathrm{meV};$ dash line:$E_{F}=30\;\mathrm{meV};$ and dash-dot line: $E_{F}=20\;\mathrm{meV}.$ Parameters used for calculation of $\sigma_{g}$ are from Ref. [21]: T=300 K and τ=1 ps..

An important question is on the controllability of amplification parameters. Figure 14 shows the normalized imaginary part of the propagation constant $k_{z}^{\left(m\right)}/k_{0}$ of the lowest mode ${\mathrm{TE}}_{y}^{\left(1\right)}$ for three different values of $E_{F}$ for the considered case $f_{\sigma }<f<f_{c}^{\left(1\right)}$. It is seen that |Im(kz(1)/k0)| is growing with $E_{F}$ although this increase has some limitations in this waveguide.

**Figure 15:**
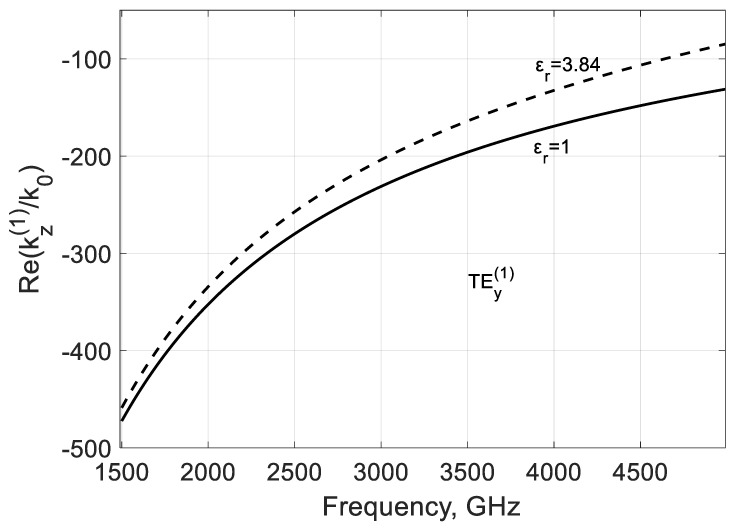
Influence of dielectric filling on the real part of propagation constant $k_{z}^{\left(1\right)}/k_{0}$ of the backward mode ${\mathrm{TE}}_{y}^{\left(1\right)}$ calculated for a graphene H-waveguide of the height b=0.01 mm, width 2a=∞, $\varepsilon_{r}=1.0;\;3.84$, and $\;\mu_{r}=1$. Parameters used for calculation of $\sigma_{g}$ are from Ref. [21]: T=300 K, $E_{F}=40\;\mathrm{meV}$, and τ=1 ps.

**Figure 16:**
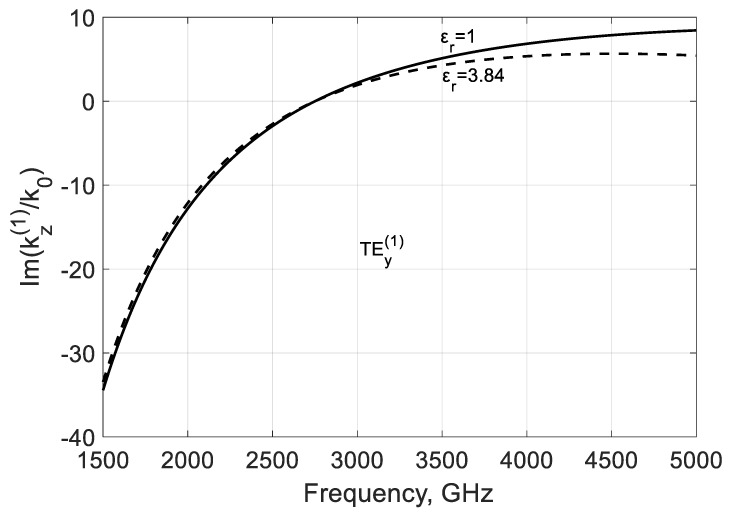
Influence of the dielectric filling on the imaginary part of the modal propagation constant $k_{z}^{\left(1\right)}/k_{0}$ of the backward mode ${\mathrm{TE}}_{y}^{\left(1\right)}$ calculated for a graphene H-waveguide of the height b=0.01 mm, width 2a=∞, $\varepsilon_{r}=1.0;\;3.84$, and $\;\mu_{r}=1$. Parameters used for calculation of $\sigma_{g}$ are from Ref. [21]: T=300 K, $E_{F}=40\;\mathrm{meV}$, and τ=1 ps.

**Figure 17:**
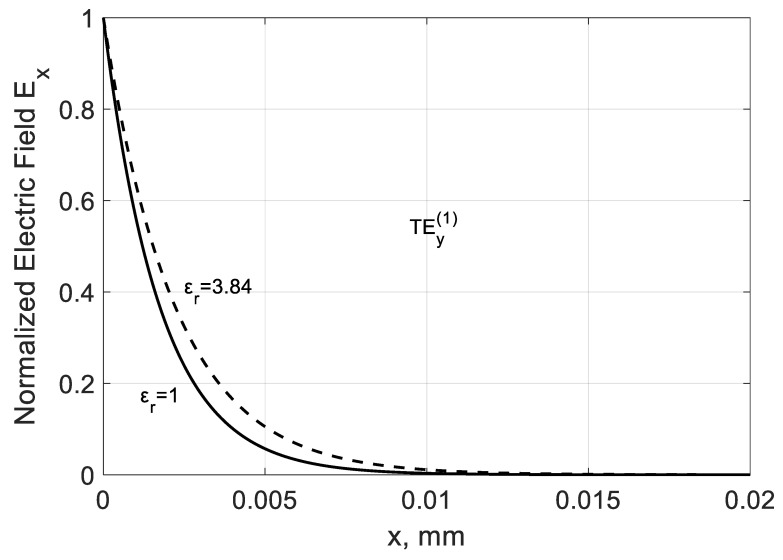
Mode field dependence $E_{x}$ along the x− direction in a graphene H-waveguide of the height b=0.01 mm and width 2a=∞ calculated for the dielectrics with $\varepsilon_{r}=1.0;3.84$ and $\mu_{r}=1$ at frequency F=4000 GHz. Parameters used for calculation of $\sigma_{g}$ are from Ref. [21]: T=300 K, $E_{F}=40\;\mathrm{meV}$, and τ=1 ps.

The modal parameters can be adjusted in part by dielectric filling. The increase in permittivity leads to some decrease in the phase β(1)=−Re(kz(1)/k0) (Figure 15) and loss $\left|\alpha^{\left(1\right)}\right|$ constants (Figure 16) as it follows from the calculations in the case $f<f_{c}^{\left(m\right)}$. Dielectric leads to partial delocalization of the field (Figure 17) and the corresponding variation of the mentioned modal parameters. 

The proposed waveguide’s guiding properties’ overall review shows that all its complex ${\mathrm{TE}}_{y}^{\left(m\right)}$ modes are amplified over $f_{\sigma }$. Waveguide shows some other interesting properties, such as the increase in amplification with the modal number for all modes below their cut-off frequencies. In opposite, over these cut-offs, the lowest mode has the largest gain.

It is worthwhile to notice that these results are obtained using the quasi-linear approach. Localization of the field close to graphene leads to increased field values, and if this field is strong enough, it influences the graphene conductivity. It limits the quasi-linear modeling’s applicability to the cases where amplification and signals are not so strong. In general, the problem should be formulated in its self-consisted nonlinear form and solved using, for instance, the iteration methods [14,15]. Another limitation is with frequency. At increased values, the conductivity shows its anisotropic property that leads to the hybridization of modes. The scalar conductivity model (12) may show some inaccuracy, and the EM theory of hybrid modes from Section 2 should be applied.

### 3.5. Conductor Loss Calculation in Graphene H-waveguide

Although the active graphene-based devices demonstrate an extremely high level of gain in order of $1000{\;\mathrm{cm}}^{-1}$ [20], the question of imperfect material use should be carefully studied. In this research, the dielectric is modeled with the complex permittivity in the Formula (8). In quartz, the dielectric loss is negligibly low compared to the level of amplification at frequencies $f_{\sigma }<f<f_{c}^{\left(1\right)}$.

Usually, in terahertz frequencies, the conductor loss prevails the dielectric one. The loss level around 10–15 dB/mm is found below 10 THz, even in a parallel-plate waveguide for ${\mathrm{TE}}_{y}^{\left(1\right)}$ propagating mode [34,51].

The conductor loss of complex modes below their cut-off frequencies is exceptionally high, and this worst-case scenario is studied below.

The conductor loss constant $\alpha_{c}^{\left(m\right)}$ of our waveguide is calculated using the perturbation approach and an enhanced Drude model of the surface resistance $R_{s}$ [8,52,53,54]. The error of this approach is within 10–20% for 2.4–12.5 THz estimated for parallel-plate waveguides. At high sub-infrared frequencies, the lossy-media wall full-wave approach is preferable [55].

An analytical formula used here to calculate the conductor loss constant $\alpha_{c}^{\left(m\right)}$ is given below

(13) αc(m)=Rs∫2L(Hm,τ)2dlRe(∫S[E×H∗]ds)=4Rs(ky(m))2(|kx(m)|2+|kz(m)|2)ωμbRe(kz(m))(κ2−(ky(m))2),

where L=(2…3)b is the integration path along with plates, b×(2…3)b is the cross-section of an area around the graphene film, Rs=Rejωμ0σD+jωε0, and Drude conductivity $\sigma_{D}=\frac{\sigma }{1+j\omega \tau_{c}}$ with $\tau_{c}$ as the mean free path time of electrons in a conductor.

**Figure 18:**
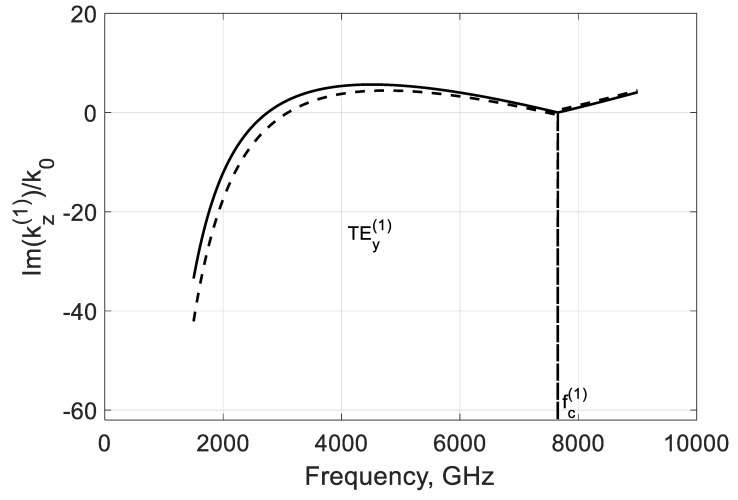
Comparison of an ideal graphene H-waveguide (solid curves) and the one having conductor loss (dash curves) for the first two backward modes. Parameters of the waveguide: b=0.01 mm, 2a=∞, $\varepsilon_{r}=3.84$, $\mu_{r}=1$, conductor conductivity $\sigma =5.96\cdot 10^{4}\;\mathrm{Sim}/\mathrm{mm}$, and $\tau_{c}=3.6\cdot 10^{14}\;s$ for copper. Parameters used for calculation of $\sigma_{g}$ are from Ref. [21]: T=300 K, $E_{F}=40\;\mathrm{meV}$, and τ=1 ps.

Figure 18 shows that, in comparison to gain, this loss can be compensated by a negative conductivity mechanism in graphene even in the worst-case scenario of the lowest mode ${\mathrm{TE}}_{y}^{\left(1\right)}$ below its critical frequency $f_{c}^{\left(1\right)}$ as it follows from our theoretical treatment. Near this frequency, the conducting loss tends to infinity (dielectric loss is not considered here) and cannot be compensated. An additional effect connected with the ${\mathrm{TE}}_{y}^{\left(m\right)}$ modes is the loss decreasing with frequency (see Formula (13) and Figure 18), known for this type of ${\mathrm{TE}}_{y}$ modes below and over their cut-off frequencies.

### 3.6. Longitudinal Field Dependence

**Figure 19:**
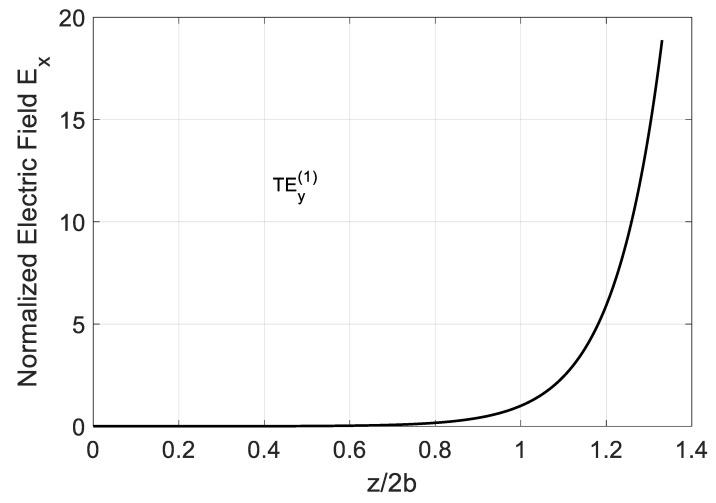
Longitudinal dependence of the transversal field $E_{x}$ of the ${\mathrm{TE}}_{y}^{\left(1\right)}$ backward mode at x=0 (graphene plane). Parameters of the graphene H-waveguide calculated at frequency f=4000 GHz: 2a=∞, b=0.01 mm $\varepsilon_{r}=1$, $\;\mu_{r}=1$, conductor conductivity $\sigma =5.96\cdot 10^{4}\;\mathrm{Sim}/\mathrm{mm}$, and $\tau_{c}=3.6\cdot 10^{14}\;s$ for copper. Parameters used for calculation of $\sigma_{g}$ are from Ref. [21]: T=300 K, $E_{F}=40\;\mathrm{meV}$, and τ=1 ps.

The found propagation constant corrected by loss in conductor plates allows calculating the field components’ longitudinal dependence along the z-axis in a more realistic manner. Figure 19 shows $E_{x}\left(z\right)$ normalized to the modal field value at z=2b. It is seen that the exponential field grows with the traveling distance.

Overall review of the obtained results shows that the proposed structure can provide a very high gain of terahertz signals, similarly to the studied earlier planar structures. Still, the impact of many other factors should be additionally investigated in the future. Among them is the influence of nonlinearity $\sigma_{g}$ at increased signal levels. The atomistic impurities and defects of graphene can presumably influence these mentioned effects. The graphene–conductor interfaces limit conductivity, and the thermal drift of all parameters can prevent increased amplification of signals. An experimental study of the proposed structure is highly desirable to establish its amplification properties reliably. After a cycle of practical research and with the maturity of the terahertz manufacturing technology, the proposed waveguide can be considered for applications in traveling-wave amplifiers of the terahertz range. To realize the lasing effect, a resonator on this waveguide should be developed. Then, the regimes of self-oscillations are found varying the parameters of waveguide and input and output loads [19,32].

## 4. Conclusions

In this paper, a novel guiding structure has been proposed for terahertz lasing applications. The waveguide consists of two parallel-plates of conductors shorted by a graphene film and composing an H-waveguide. The negative conductivity of graphene is induced by pumping light, and the THz modes are amplified propagating along with this structure. At the difference to known designs, the proposed waveguide can have decreased loss due to the complete exclusion of the sharp-edged conductors and graphene edged strips from its design and using the ${\mathrm{TE}}_{y}^{\left(m\right)}$ modes with the electric fields falling towards conducting plates. The EM field’s better concentration near graphene is provided, employing a geometry evanescent for the waves propagating away from this film.

This waveguide has been analyzed in a quasi-linear regime using a rigorous EM method of field matching. The algorithm is formulated for the hybrid, ${\mathrm{TE}}_{y}$, and ${\mathrm{TM}}_{y}$ modes, and the two last of them are calculated analytically. The modal-type selective amplification has been discovered, and the ${\mathrm{TE}}_{y}$ modes have been studied in detail. It has been found that the proper ${\mathrm{TE}}_{y}^{\left(m\right)}$ modes being bound at frequencies of negative resistance of graphene can be guided and amplified in this waveguide even considering the parallel-plate conductor loss calculated in Drude approximation.

The waveguide’s design can be enhanced further using the multilayered graphene conductors and heterostructures to increase pumping light absorption [27,56]. It has been noticed on the necessity of further research of this promising waveguide to establish the limits of accuracy of the developed model taking into account, for instance, the nonlinearity of graphene conductivity at strong signals, its anisotropy at increased terahertz (>10 THz) frequencies, and the influence of technological factors on the output parameters [15].

The obtained results highlight the EM interaction mechanisms with graphene films showing the modal-type and mode-dependent amplification and irradiation in the studied open graphene-based structure. Based on our theory, a set of waveguides and components can be designed for the loss-compensated interconnections and active antennas. Self-oscillating devices can be developed by creating the active resonators in which the instability condition is provided by choosing the proper geometry of a proposed H-waveguide and loads [19].

## References

[1] Geim A.K., Novoselov K.S. (2007). The rise of graphene. Nat. Mater..

[2] Heydari M.B., Samiei M.H.V. (2018). Plasmonic graphene waveguides: A literature review. arXiv.

[3] Gusynin V., Sharapov S., Carbotte J. (2006). Magneto-optical conductivity in graphene. J. Phys. Cond. Matt..

[4] Falkovsky L.A., Varlamov A.A. (2007). Space-time conductivity of graphene. Eur. Phys. J. B.

[5] Dubinov A.A., Aleshkin V.Y., Mitin V., Otsuji T., Ryzhii V. (2011). Terahertz surface plasmons in optically pumped graphene structures. J. Phys. Cond. Matt..

[6] Lovat G., Hanson G.W., Araneo R., Burghignoli P. (2013). Comparison of spatially dispersive models for dyadic intraband conductivity of graphene. Proceedings of the 2013 7th European Conference on Antennas and Propagation (EuCAP).

[7] Hanson G.W. (2008). Quasi-transverse electromagnetic modes supported by a graphene parallel-plate waveguide. J. Appl. Phys..

[8] Kouzaev G.A. (2019). Physics-based analytical engineering models of graphene micro- and nanostrip lines. IEEE Trans. Compon. Packag. Manuf. Technol..

[9] Lovat G., Burghignoli P., Araneo R. (2013). Low-frequency dominant mode propagation in spatially dispersive graphene nanowaveguides. IEEE Trans. Electromagn. Compat..

[10] Lovat G., Ye D., Burghignoli P., Araneo R., Wei X.-C. (2018). Theoretical study of the first higher order mode in grounded graphene nanoribbons. IEEE Trans. Nanotech..

[11] Shao Y., Yang J.J., Huang M. (2016). A review of computational electromagnetic methods for graphene modeling. Int. J. Antennas Propag..

[12] Yang J., Hu P., Yu G. (2019). Perspective of graphene-based electronic devices: Graphene synthesis and diverse applications. APL Mater..

[13] Otsuji T., Tombet S.A.B., Satou A., Fukidome H., Suemitsu M., Sano E., Popov V., Ryzhii M., Ryzhii V. (2012). Graphene-based devices in terahertz science and technology. J. Phys. D.

[14] Makeeva G.S., Golovanov O.A., Kouzaev G.A. (2017). Numerical analysis of tunable parametric terahertz devices based on graphene nanostructures using the projection method and autonomous blocks. Proc. AIP Conf..

[15] Lerer A.M., Makeeva G.S., Kouzaev G.A. Electrodynamic and probabilistic calculation of performances of THz devices based on periodic multilayer graphene-dielectric structures. Proceedings of the Moscow IEEE Workshop on Electronic and Networking Technologies (MWENT).

[16] Ooi K.J.A., Tan D.T.H. (2017). Nonlinear graphene plasmonics. Proc. R. Soc. A.

[17] Bozzi M., Pierantoni L., Bellucci S. (2015). Application of graphene at microwave frequencies. Radioengineering.

[18] Ryzhii V., Ryzhii M., Otsuji T. (2007). Negative dynamic conductivity of graphene with optical pumping. J. Appl. Phys..

[19] Rana F. (2008). Graphene terahertz plasmon oscillators. IEEE Trans. Nanotechnol..

[20] Ryzhii M., Ryzhii V. (2007). Injection and population inversion in electrically induced p–n junction in graphene with split gates. Jpn. J. Appl. Phys..

[21] Popov V.V., Polischuk O.V., Davoyan A.R., Ryzhii V., Otsuji T., Shur M.S. (2012). Plasmonic terahertz lasing in an array of graphene nanocavities. Phys. Rev. B.

[22] Ryzhii V., Dubinov A.A., Otsuji T., Mitin V., Shur M.S. (2010). Terahertz lasers based on optically pumped multiple graphene structures with slot-line and dielectric waveguides. J. Appl. Phys..

[23] Chai J., Hu P., Ge L., Xiang H., Han D. (2019). Tunable terahertz cloaking and lasing by the optically pumped graphene wrapped on a dielectric cylinder. J. Phys. Commun..

[24] He X.Q., Ning T.G., Pei L., Zheng J.J., Li J., Wen X.D. (2019). Tunable hybridization of graphene plasmons and dielectric modes for highly confined light transmit at terahertz wavelength. Opt. Express.

[25] Ryzhii V., Ryzhii M., Sato A., Otsuji T., Dubinov A.A., Aleshkin V.Y. (2009). Feasibility of terahertz lasing in optically pumped epitaxial multiple graphene layer structures. J. Appl. Phys..

[26] Kumbhare V.R., Paltani P.P., Majumder M.K. (2018). Future of graphene based interconnect technology—A reality or a distant dream. Proceedings of the 2018 5th IEEE Uttar Pradesh Section International Conference on Electrical, Electronics and Computer Engineering (UPCON).

[27] Morozov M.Y., Leiman V.G., Popov V.V., Mitin V., Shur M.S., Karasik V.E., Ryzhii M., Otsuji T., Ryzhii V. (2019). Optical pumping in graphene-based terahertz/far-infrared superluminescent and laser heterostructures with graded-gap black-P_x_As_1-x_ absorbing-cooling layers. Opt. Eng..

[28] Lee K.W., Jang C.W., Shin D.H., Kim J.M., Kang S.S., Lee D.H., Kim S., Choi S.-H., Hwang E. (2015). Light-induced negative differential resistance in graphene/Si-quantum-dot tunneling diodes. Sci. Rep..

[29] Li H., Yan M., Wan W., Zhou T., Zhou K., Li Z., Cao J., Yu Q., Zhang K., Li M. (2019). Graphene-coupled terahertz semiconductor lasers for enhanced passive frequency comb operation. Adv. Sci..

[30] Ryzhii V., Otsuji T., Shur M. (2020). Graphene based plasma-wave devices for terhahertz applications. Appl. Phys. Lett..

[31] Quispe H.O.C., Encomendero-Risko J.J., Xing H.G., Sensale-Rodriguez B. (2016). Terahertz amplification in RTD-gated HEMTs with a grating-gate wave coupling topology. Appl. Phys. Lett..

[32] Mao X., Xie S., Zhu C., Geng Z., Chen H. (2018). Theoretical study of terahertz active transmission line oscillator based on RTD-gated HEMT. AIP Adv..

[33] Ahi K. (2017). Review of GAN-based devices for terahertz operation. Opt. Eng..

[34] Mitrofanov O., James R., Fernández F., Mavrogordatos T.K., Harrington J.A. (2011). Reducing transmission losses in hollow THz waveguides. IEEE Trans. Terahertz Sci. Technol..

[35] Tischer F.J. (1959). Properties of the H-guide at microwave and millimeter-wave regions. Proc. IEE Part B Electron. Commun. Eng..

[36] Yoneyama T., Nishida S. (1981). Non-radiative dielectric waveguide for millimeter-wave integrated circuits. IEEE Trans. Microw. Theory Tech..

[37] Kuroki F., Ohta H., Yoneyama T. (2005). Transmission characteristics of NRD guide as a transmission medium in THz frequency band. Proceedings of the 2005 Joint 30th International Conference on Infrared and Millimeter Waves and 13th International Conference on Terahertz Electronics.

[38] Ye L., Xu R., Wang Z., Lin W. (2010). A novel broadband coaxial probe to parallel plate dielectric waveguide transition at THz frequency. Opt. Express.

[39] Sesao K., Monnai Y. (2020). Variable terahertz attenuator integrated on non-radiative guide using photoinduced carriers. IEEE Trans. Terahertz Sci. Technul..

[40] Meixner J. (1972). The behavior of electromagnetic fields at edges. IEEE Trans. Antennas Propag..

[41] Balandin A.A. (2011). Thermal properties of graphene and nanostructured carbon materials. Nat. Mater..

[42] Balanis C.A. (1989). Advanced Engineering Electromagnetics.

[43] Witt H.R., Biss R.E., Price E.L. (1968). Propagation constants of a waveguide containing parallel sheets of a finite conductivity. IEEE Trans. Microw. Theory Tech..

[44] Felsen L.B., Marcuvitz N. (1973). Radiation and Scattering of Waves.

[45] Hanson G.W. (2008). Dyadic Green’s function and guided surface waves for a surface conductivity model of graphene. J. Appl. Phys..

[46] Mohadesi V., Siahpoush V., Asgari A. (2017). Investigation of leaky and bound modes of graphene surface plasmons. J. Appl. Phys..

[47] Shvechenko V.V. (2007). Forward and backward waves: Three definitions and their interrelation and applicability. Phys. Uspekhi.

[48] Santos A.M., Beliaev D., Scolfaro L.M.R., Leite J.R. (1999). Quasi-Fermi levels, chemical and electric potentials profiles of a semiconductor under illumination. Braz. J. Phys..

[49] Monticone F., Alu A. (2015). Leaky-wave theory, techniques, and applications: From microwaves to visible frequencies. Proc. IEEE.

[50] Xu F., Wu K. Understanding leaky-wave structures. IEEE Microw. Mag..

[51] Mendis R., Mittleman D.M. (2009). Comparison of the lowest-order transverse-electric (TE1) and transverse-magnetic (TEM) modes of the parallel-plate waveguide for terahertz pulse applications. Opt. Express.

[52] Kouzaev G.A. (2013). Applications of Advanced Electromagnetics. Components and Systems.

[53] Lucyszyn S. (2003). Accurate CAD modelling of metal conduction losses at terahertz frequencies. Proceedings of the 11th IEEE International Symposium on Electron Devices for Microwave and Optoelectronic Applications, EDMO 2003.

[54] Lucyszyn S. (2007). Evaluating surface impedance models for terahertz frequencies at room temperature. PIERS Online.

[55] Yeap K.H., Tham C.Y., Yassin G., Yeong K.C. (2011). Attenuation in rectangular waveguides with finite conductivity walls. Radioengineering.

[56] Li M.-Y., Chen C.-H., Shi Y., Li L.-J. (2016). Heterostructures based on two-dimensional layered materials and their potential applications. Mater. Today.

